# Structural and Functional Changes in Aging Kidneys

**DOI:** 10.3390/ijms232315435

**Published:** 2022-12-06

**Authors:** Jill Dybiec, Magdalena Szlagor, Ewelina Młynarska, Jacek Rysz, Beata Franczyk

**Affiliations:** Department of Nephrology, Hypertension and Family Medicine, Medical University of Lodz, Ul. Żeromskiego 113, 90-549 Łódź, Poland

**Keywords:** aging, kidney function, elderly, nephrosclerosis, glomerular filtration rate

## Abstract

The renal condition is one of the crucial predictors of longevity; therefore, early diagnosis of any dysfunction plays an important role. Kidneys are highly susceptible to the aging process. Unfavorable conditions may lead to a significant disturbance of the body’s homeostasis. Apart from physiological changes, there are some conditions such as hypertension, diabetes or obesity which contribute to the acceleration of the aging process. A determination of macroscopic and microscopic changes is essential for assessing the progression of aging. With age, we observe a decrease in the volume of renal parenchyma and an increase in adipose tissue in the renal sinuses. Senescence may also be manifested by the roughness of the kidney surface or simple renal cysts. The main microscopic changes are a thickening of the glomerular basement membrane, nephrosclerosis, an accumulation of extracellular matrix, and mesangial widening. The principal aspect of stopping unfavorable changes is to maintain health. Studies have shown many useful ways to mitigate renal aging. This review is focused especially on medications such as renin-angiotensin-aldosterone system blockers or resveratrol, but even eating habits and lifestyle.

## 1. Introduction

Aging is a process associated with a range of damage both on a molecular and cellular level. The first age-related body changes appear after the age of 30. They include the loss of bone, muscle mass, and cartilage, an increase of fat, the alteration of hormone levels, and many others [1]. The renal condition seems to be one of the most crucial predictors of longevity [2]. The key role of the kidneys is to remove waste products from the blood and also regulate the levels of many essential compounds. Chronic kidney disease is one of the major causes of death worldwide, as well as a leading cause of years of life lost [3]. Due to the kidney’s high susceptibility to senescence, early detection of any dysfunction is very important for survival. However, maintaining health is definitely more crucial. Studies have shown that resveratrol or renin-angiotensin-aldosterone system blockers can be helpful to mitigate renal aging. Yet, something that seems to be the most beneficial for renal function is simple habits such as appropriate diet and exercise. 

### 1.1. The Role of the Kidney in the Human Body

Every minute, the kidneys filter 800–1200 mL of blood, removing the waste products of metabolism and excessive fluid from the body, generating ca. 1.5 L of urine. They are also responsible for the maintenance of the water–electrolyte balance and blood pressure. It is worth adding that the kidneys are also hormonally active, secreting hormones such as erythropoietin, renin, and calcitriol. The physiology of the kidneys is associated with very essential metabolic procedures, and if placed under unfavorable conditions, such as excessive and prolonged oxidative stress, kidneys may lose their regular function and the homeostasis of the organism may be disturbed. Further, kidneys are highly susceptible to the aging process [4]. Given the above, early diagnosis of renal dysfunction and therapy implementation are crucial to survival. 

### 1.2. Loss of Renal Function

Aging is a genetically determined, physiological process in every organism, which affects structural and functional changes within all systems. The kidneys are one of the organs most prone to aging [5], which is manifested, for example, as a decrease in the number of nephrons with age and glomerular filtration. These natural changes can be influenced by non-modifiable factors, such as sex or race, and a number of comorbidities that people struggle with throughout their lifetime. Most of them are risk factors that can have an impact on the development of these renal changes. The presence of diseases, such as hypertension (HT), diabetes, obesity and more, can accelerate the aging of the kidneys [6]. It is worth adding that most potentially healthy elderly people will only have minimal progression of kidney disease [7], and that comorbidities have the greatest impact on the progression of the kidney disease and aging.

The primary measurement used to estimate renal condition is total glomerular filtration rate (GFR). The Kidney Disease Improving Global Outcome organization (KDIGO) recommended this test as one of the best methods for the early diagnosis of Chronic Kidney Disease (CKD) [8]. In 1950 Davis et al. reported a linear decline of GFR in healthy adults when they reach the age of 30 [9]. Moreover, by the age of 90, GFR was reduced by 46% from that detected at the beginning of the study. Bolignano et al. reported that the annual average reduction in GFR is about 0.4 to 2.6 mL/min/year [10]. This decline is attributable to nephron loss. Nevertheless, this assumption became a controversial issue because of the disproportion of the reduction in GFR and the number of nephrons. However, Denic et al. concluded that glomeruli can undergo sclerosis and atrophy in the aging process and then be resorbed [11].

Due to Fliser’s research, it was suggested that one of the major reasons for the age-related decline in GFR are changes in renal hemodynamics [12,13]. It is worth mentioning that this decline can be also correlated with decreased protein intake, which is common among elderly people [14].

## 2. The Features of Kidney Aging

### 2.1. Macroscopic Changes in the Kidneys 

The determination of macroscopic changes in the kidneys is one of the key elements in assessing the progression of changes in an aging organism. An estimation can be made by autopsy or imaging tests, such as ultrasound or computed tomography.

Autopsy allows for an accurate macroscopic evaluation of aging kidneys, but it should be remembered that the main disadvantage of this method is the possibility of detecting kidney disease that may have contributed to the patient’s death, which may lead to an erroneous assessment of kidney development with age. Based on many autopsy studies, it has been estimated that from about 4–5 to 7–8 decades of life, the kidney mass decreases by 10–30%. The reduction of kidney mass in an aging organism is also confirmed by imaging studies in patients [15].

Imaging studies in elderly patients showed a decrease in the volume of renal parenchyma with age, both in the case of people with comorbidities [15] and without them [16]. Total kidney volume decreases by about 16 cm3 per decade, although most of the decline occurs after the age of 60 years old [17]. In addition, autopsy studies revealed a lower decline in kidney volume with age, independently of comorbidities. While no significant changes in kidney volume were observed in CT with age in patients who were potential kidney donors [15], a study by Wang X et al. [18], using the CT data of 1334 living kidney donors, showed that kidney volume decreased with age after 50 years of age. Until the age of 50, the volume of the cortical layer decreases, which is associated with a compensatory increase in the medullary volume.

The reduction in the volume of the renal parenchyma is also compensated by the increase in the volume of adipose tissue in the renal sinuses [8]. Excess fat tissue in the renal sinuses, the so-called “fatty kidney”, may be a risk factor for the development of arterial HT [19].

Other macroscopic changes in the kidneys, the incidence of which increases with age, are renal artery fibromuscular dysplasia, atherosclerotic narrowing, focal cortical thinning, parenchymal calcification, and the prevalence of indeterminate masses [15,20]. The aging of the kidneys is also manifested by the roughness of the kidney surface [10,20]. Renal tubular diverticula appearing in the aging process may cause simple renal cysts [21], the frequency of which also increases with age [15,17]. Renal cysts can occur in one or both kidneys. They are usually clinically insignificant; however, studies have shown that their presence may correlate with reduced kidney size [22] or arterial HT [23].

### 2.2. Histological Changes

The main microscopic changes that characterize renal aging are the thickening of the glomerular basement membrane, nephrosclerosis [24], the accumulation of extracellular matrix, and mesangial widening [25]. Nephrosclerosis is defined as focal global glomerulosclerosis, arteriosclerosis, tubular atrophy, and interstitial fibrosis [15]. These findings are particularly characteristic for HT [26], but they also occur in healthy individuals [27]. The prevalence of nephrosclerosis increased linearly from 2.7% for healthy adults aged 18 to 29 years to 73% for those aged 70 to 77 years.

Randles et al. reported that in mice models of kidney disease, the extracellular matrix changes with age. There is an accumulation of interstitial matrix proteins, such as collagens I, III, VI, XV, fibrinogens, as well as nephronectin, and a reduction of basement membrane components, such as laminins, type IV collagen, and type XVIII collagen [28].

### 2.3. Molecular Changes

Cellular senescence, a stress response and major cause of aging, is characterized by permanent cell-cycle arrest, resistance to apoptosis, and senescence-associated secretory phenotype (SASP) [4]. SASP is an important factor which recruits immune cells required for senescent-cell clearance [29]. Consequently, the impairment of this process can result in the progressive accumulation of chronic senescent cells and therefore lead to a loss of cells responsible for repair and regeneration. SASP can be a very crucial factor in antiaging treatment; for example, Wang et al. investigated that in order to mitigate renal fibrosis which occurs in the aging kidney, multipharmacological approaches focusing on several SASP are needed [30]. 

The main pathways involved in renal ageing are p53/p21 and p16/Rb [31]. DNA damage initiates the activation of signaling cascades (ATM, ARF, or the p53 network). This mechanism contributes to the increased expression of p21CIP1 and p16INK4a and leads to the inhibition of phosphorylation of CDK complexes and retinoblastoma protein (Rb) [32]. This results in the inhibition of E2F activity by Rb which ultimately stops cell proliferation and ends in renal senescence. It is noteworthy that due to their different functions, kidney cells manifest different hallmarks of cellular senescence which are presented in Table 1. The activity of p16INK4a, as a senescence mediator, may also be induced by oxidative stress, oncogenic Ras expression, and epigenetic alterations [33]. Some publications suggest the presence of p16INK4a, prior to morphologic changes which entail cell senescence, participates in the pathogenesis of renal diseases [34]. Interestingly, Chen et al. [35] showed that 1,25(OH)2 D3 plays a significant role in this pathway. Among others, it is responsible for delaying aging by inactivating p53-p21 and p16-Rb signaling pathways.

Senescence is determined by telomere shortening, decreased Klotho expression, and the inactivation of silent information regulator T1 (SIRT1). Lim et al. showed that oxidative stress mediated by sirtuin 1 (SIRT1), PGC-1α, PPARα, and Klotho can be a cause of tissue injury in the aging process [40].

The expression and activity of SIRT1 decrease with the age. Reduced SIRT1 effects PGC-1 which may exacerbate oxidative stress. Suppression of SIRT1/PGC-1α signaling may also lead to catabolism, angiogenesis, inflammation, and mitochondrial dysfunction [41]. Nevertheless, SIRT1 can be activated by calorie restriction and exercise which seems to be an important issue for health maintenance [42]. Moreover, Ryu et al. demonstrated that SIRT1-induced deacetylation of HIF-1α (transcription factor responsible for adaptive cellular responses to hypoxia) can protect interstitial tubules from age-related injury [43]. This finding raises therapeutic hopes for slowing damage in aging kidneys.

Klotho is a gene that is principally expressed in the renal distal tubular cells. It is known for regulating phosphate excretion, but most importantly for its “anti-aging” effect. The anti-aging properties of Klotho are the result of the stabilization of the NFκB–IKK complex and the inhibition of NFκB translocation from the cytoplasm to the nucleus [44]. There are two types of Klotho protein—secreted and membrane. Secreted Klotho is a humoral factor responsible for regulating the activity of several glycoproteins. Membrane Klotho is a co-receptor for FGF23, a hormone that is responsible for phosphate excretion into urine. Isakova et al. reported that increased FGF23 levels were linked with higher mortality [45]. This finding revealed a connection between phosphate metabolism and senescence. A Western diet which is especially high in phosphate has been shown to increase levels of FGF23, and hence decrease Klotho [46]. Animals with genetically determined klotho deficiency show accelerated aging and a progressive decline in renal function [47]. In contrast, overexpression of Klotho increases lifespan in mice by 20–30% [48].

Liu et al. [49] conducted a meta-analysis in which they indicated the important role of Klotho for patients with CKD. Due to the positive correlation between levels of sKlotho and eGFR, they proposed to use sKlotho as a novel biomarker in the early detection of the deterioration of renal function. Interestingly, sKlotho supplementation reduces blood pressure and albuminuria by increasing renal angiotensinogen and angiotensin II levels [50]. Nevertheless, by inhibiting TGF-β and TNF signaling and suppressing endoplasmic reticulum stress or epithelial-mesenchymal transition, sKlotho reduces renal fibrosis [51,52,53]. Due to its beneficial properties, the administration of sKlotho seems to be a novel kidney-protective management method, especially for renal fibrosis since it is well known that aged kidneys present remarkably increased fibrosis. Recent studies have revealed that Klotho levels can be restored to normal or upregulated [54]. The restoration of endogenous Klotho might be a new significant direction in kidney disorder therapy. One of the major epigenetic mechanisms that affect Klotho’s expression in renal dysfunction is DNMT and HDAC enzymes. Therefore, the inhibition of this process is an essential element for maintaining health. Fortunately, the FDA has already approved a few DNMT and HDAC inhibitors in oncology [55]. However, due to the potential cytotoxicity of these drugs in long-term use [56], a lot of work needs to be completed to evaluate their efficacy and safety. Yet, it is worth mentioning that commonly used agents such as statins or thiazolidinediones are also capable of increasing Klotho expression.

The activity of the nuclear peroxisome proliferator-activated receptor-γ (PPARγ), which decreases with age, is also crucial in the kidney aging process. The PPARγ pathway reduces vascular oxidative stress, while PPARγ agonists increase Klotho expression [57]. Retrospective studies suggest that increased PPARγ activity prevents proteinuria [58], which physiologically increases with aging. Therefore, the use of PPARγ agonist antidiabetics, such as pioglitazone, should be considered in the therapeutic strategy of elderly patients with reduced PPARγ activity. Prospective studies in this direction are critical. 

In Wung Chung et al. study [59], researchers investigated peroxisome proliferator–activated receptor α (PPARα) and the fatty acid oxidation (FAO) pathway as regulators of age-associated renal fibrosis. Along with the accumulation of lipids in the renal tubular epithelial region during aging in rats, the expression of PPARα and the FAO pathway-associated proteins decreased. Responsible for these changes during aging is the increased expression of PPARα-targeting microRNAs, especially miR-21. The results of this study conclude that defects in renal PPARα signaling during aging aggravates renal fibrosis development and that targeting PPARα may be useful for preventing age-associated CKD.

Another factor that participates in the renal aging process is hyperglycemia. Wu et al. reported that hyperglycemia-related advanced glycation end products (AGEs) are the main component in the pathophysiology of DKD [60]. The activation of the AGEs-mediated receptor for AGEs (RAGE) can initiate the production of reactive oxygen and nitrogen species and therefore elicit oxidative stress in the kidney. Hence, one of the intervention strategies for DKD seems to be targeting RAGE and its ligands. Examples of pharmacotherapeutic targets are presented in Table 2. 

## 3. Kidney Diseases

It should also be remembered that renal aging itself is a risk factor for the occurrence of many disorders of the kidneys and other organ systems [31].

### 3.1. Acute Kidney Injury

The elderly are at risk of Acute Kidney Injury (AKI). The decline of nephron numbers and GFR in aged kidneys could result in an increased susceptibility to AKI [31]. In aging kidneys, phosphate, glucose and sodium reabsorption are impaired, as well as the ability to concentrate and dilute urine. Insufficient fluid intake, diarrhea, or vomiting leading to dehydration, combined with disturbances in the fluid and electrolyte balance may contribute to the development of acute renal failure.

Advanced age also predisposes to AKI which requires dialysis, because older people suffering from AKI go through it more severely [4]. Moreover, they have decreased kidney repair after AKI given the high incidence of progression to CKD [67].

### 3.2. Chronic Kidney Disease 

In an aging body, it comes following a progressive reduction in glomerular filtration in the kidneys, and albuminuria. According to the levels of GFR and albuminuria based on the KDIGO 2012 clinical practice guidelines, CKD can be diagnosed. CKD is present if the patient has an estimated glomerular filtration rate (eGFR) less than 60 mL/min/1.73 m^2^ that persists for 3 months or longer, even in the absence of any confirmatory findings of kidney damage [20]. 

In CKD, water and electrolyte balance disorder occur resulting in, e.g., overhydration and hyperkalemia. Furthermore, waste products that accumulate in the body as uremic toxins are not excreted properly. Elimination disorders also impair the excretion of xenobiotics and drug metabolites, leading to their accumulation in the body. CKD also results in a disturbance in the regulation of calcium and phosphate metabolism. Vitamin D3 deficiency, as well as hyperphosphatemia, caused by impaired renal function, results in hypocalcemia-accelerating secondary hyperparathyroidism. CKD also caused hypogonadotropic hypogonadism by reducing the secretion of gonadotrophins, resulting in amenorrhea or impotence. A number of these abnormalities, especially if left untreated, can lead to premature death.

However, Chia-Tse Yeh et al.’s research suggests that age is also a crucial modifier in CKD progression. Elderly people over 65 years old with moderate to advanced CKD could possess a decreased risk of end-stage kidney disease in comparison to younger patients [68].

## 4. Factors That May Be Deeply Related to Kidney Aging

### 4.1. Non-Modifiable Risk Factors

There are a lot of studies confirming the influence of sex on the aging of the kidneys [69,70]. Based on study result, it can be stated that estrogens have a beneficial, protective effect on renal function in the elderly, while androgens have the opposite effect [69,71]. It suggests that male sex is a determinant of kidney function decline [72]. The gradual decline in testosterone level experienced in older adults may contribute to age-related kidney dysfunction. 

Prostaglandin E2 levels also declines with age in both men and women. A Gava et al. study reported that high levels of prostaglandin E2 correlate with greater resistance to the progression of kidney disease in women [69]. Moreover, in most postmenopausal women, testosterone levels increase as renal failure develops and cardiovascular risk increases, suggesting that high levels of this hormone in the blood are harmful and destructive. In men, chronic renal failure progresses faster than in women implying that gender is one of the determinants of renal disease progression with age [69].

Race is also an essential factor in damaging the kidneys and thus accelerating aging. African Americans are at particular risk of the hardening of their kidneys from HT [73]. The reduction in the number of nephrons causes glomerular hypertrophy, and hyperfiltration, leading to HT with the consequent glazing of the glomeruli. Glomerular changes, such as fibrosis and glazing, are significantly more common in African Americans than in Caucasians and may account for a higher incidence of hypertensive nephropathy in black people [72].

### 4.2. Modifiable Risk Factors

It is difficult to distinguish between physiological renal aging and disease-mediated changes in structure and function that are more common in the elderly. Nevertheless, it is important to emphasize that age-related diseases, when overlapping with those related to normal aging, may affect the rate of deterioration of function, exhaust the functional reserve of the kidneys, and predispose these patients to kidney damage [20]. Cardiovascular diseases (CVD), HT, smoking, and diabetes were identified as determinants of kidney function decline [70]. It is not yet clear to what extent the loss of kidney function is only related to age and to what is associated with CVD and exposure to risk factors such as high blood pressure, diabetes, and smoking throughout life [73].

### 4.3. Cardiovascular Diseases

The age-related loss of renal function is more pronounced in older people with CVD [6,74]. In people with cardiovascular diseases, we observe progressive changes in the vessels, such as endothelial dysfunction by smoothing the intima of the arteries and vascular wall calcifications, and thus progressive arteriosclerosis [74]. All these changes also take place in the kidney vessels, disrupting normal blood flow through the kidneys and causing their progressive, faster-than-physiological dysfunction, called nephrosclerosis. These changes are also characteristic of ischemic nephropathy, which in the elderly is usually caused by microvascular atherosclerosis [75]. Due to the development of systemic atherosclerotic changes in the kidneys, cholesterol embolism, microinfarcts, and the formation of parenchymal scarring may occur [71]. Disturbances in renal blood flow, as well as a progressive decrease in the number of nephrons, result in a decline in glomerular filtration in the kidneys and accelerating aging, as well as leading to CKD. Moreover, the associated comorbidities and ensuing vascular disease may also determine mortality in elderly individuals with CKD [73].

### 4.4. Hypertension

Hypertension is one of the most common chronic diseases in Poland. According to Polish epidemiological studies conducted from 1997 to 2017, arterial HT occurs in 29% (NATPOL PLUS) to 45% (WOBASZ II) of the adult population and even in 75% of people aged 65 years and above (PolSenior) [76]. It is worth adding that elevated systolic blood pressure is a risk factor for many diseases and pathologies, particularly age-associated decline in renal function in older people [6,74].

Chronic HT, especially untreated, in combination with atherosclerosis, age, or smoking [6,70,74], can cause hypertensive nephropathy which also manifests as vessel sclerosis. This damage impedes renal function which leads to their faster aging and insufficiency. The reduction in systolic blood pressure in people over 65 could result in the decreased risk of these changes [6]. Moreover, the associated comorbidities and ensuing vascular disease may also determine mortality in elderly individuals with CKD [74].

### 4.5. Tobacco

Longitudinal studies show that decline in renal function in the elderly is also associated with tobacco [70,74]. Heavy current smoking, being responsible for large and small vessel disease, is highly associated with clinically important renal changes in an older population which may lead to accelerated aging of the kidneys.

Furthermore, smoking has an impact on CKD progression in patients [77,78], especially diagnosed with renal deterioration [79]. High glomerular filtration rate and greater prevalence of proteinuria in smokers suggests a hyperfiltration mechanism. These factors might result in renal damage and further CKD [80].

Anthony J. Bleyer’s research indicates that the cessation of tobacco usage and smoking could decrease morbidity of renal insufficiency in the elderly [6]. Smoking discontinuance improves circulatory system parameters leading to a regression in microalbuminuria, which may cause a reduction in CKD progression in diabetics [79].

### 4.6. Body Mass Index

Overweight and obesity are a growing health problem in the general population. Excessive obesity raises blood pressure and is one of the main causes of cardiovascular and kidney diseases. Obese patients show an increase in renal plasma flow, filtration fraction and more frequent incidences of proteinuria. With the duration and severity of obesity, the risk of impaired renal function increases.

Elevated body mass index, waist circumference, and waist-to-height ratio have been shown to be risk factors for GFR decline and death in people who have normal or decreased levels of estimated GFR [81]. Obesity also correlates with abnormal kidney function and focal segmental glomerulosclerosis, suggesting that elevated Body Mass Index (BMI) may be a cause of chronic renal failure, leading to its further progression and faster kidney aging. Obese people have more than twice the risk of CKD than physically active people [78]. High BMI level is a risk factor for nephrolithiasis and kidney cancer [82].

Despite this, it is worth noting that obesity does not increase the risk of end-stage renal disease (ESRD) in patients with moderate to advanced CKD [83].

### 4.7. Insulin Resistance and Diabetes Mellitus

A prospective cohort study of Hui-Teng Cheng et al. [84] revealed that insulin resistance is affiliated with prevalent CKD and is associated with a rapid decline in renal function and further aging. Impaired insulin sensitivity results in the altered renal cell metabolism of glucose [85]. Moreover, the researchers stated that metabolic syndrome predicts the risks of prevalent and incident CKD [84]. 

Diabetes mellitus, similar to insulin resistance, is also identified as a modifiable risk factor for kidney function impairment and an increase in the aging process. Renal accelerated aging in people with diabetes mellitus (DM) correlates with the accumulation of AGEs, oxidative stress, and inflammation. It is also associated with HT [86,87]. In patients with diabetes, De Burgh et al. [70] detected higher baseline eGFR, but faster eGFR decline with age, and a detrimental impact on the albumin-to-creatinine ratio (ACR), whereas prediabetes was identified as determinative for the ACR increase only, and the effect on eGFR was insignificant. Based on that data, it can be assumed that prediabetes causes selective damage to the glomerular filtration barrier which does not yet affect kidney function. Implementing appropriate treatment for prediabetes can prevent the later loss of kidney function. In cases of patients who suffer from DM, new anti-diabetic, sodium-glucose cotransporter-2 (SGLT2) inhibitors have significant value in slowing down the progression of kidney disease in patients with type 2 diabetes [88].

### 4.8. High Protein Intake

Another factor affecting the functioning of the kidneys is high protein intake, which may cause increased blood pressure and circulation in the capillaries of the glomeruli, which can lead to glomerular injury and proteinuria [89]. All these changes may result in renal hyperfiltration, progressive hardening of the kidneys, and thus age-related decreased renal function [90].

The protein source and quality may be vital. High red-meat consumption might increase CKD risk, whereas fruit and vegetable proteins may be a renal protective factor. Furthermore, poultry and dairy CKD risk is insignificant [91]. 

Despite some findings, research into protein intake and its effects on kidney function and aging is still too low to conclude the negative effect of long-term, high-protein intake. The results of the available studies are contradictory and do not allow for unambiguous conclusions.

**Figure 1:**
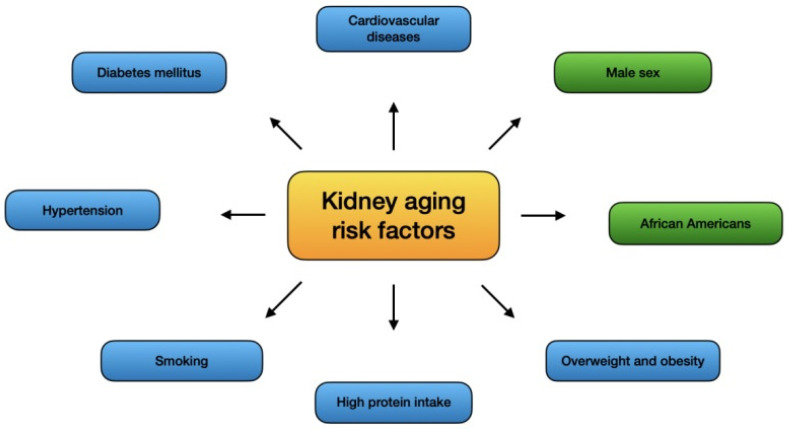
Risk factors of kidney aging. 1.

Described risk factors are presented in Figure 1.

## 5. Treatment

### 5.1. Kidney Transplantation

Currently, there is a small proportion of older dialysis patients who are eligible for kidney transplantation. It may be due to medical contraindications, long waiting times, or a limited donor pool. However, studies have evidently shown that this group of patients does benefit from this treatment path.

In 1996, Laupacis et al. conducted a study in which they compared the utility of renal transplantation with dialysis [92]. A cohort of 168 patients was observed for about two years after transplantation. Health-related quality of life was measured by questionnaires. It was found that after-surgery life satisfaction indicators improved for the entire cohort. The utility of dialysis was initially 0.55 for patients 60 year old and older and then increased to 0.72 with a kidney transplant. 

Another study in which the mean age of subjects was 64.8 revealed that transplantation was seen by the patients as an opportunity to age successfully [93]. Moreover, Humar et al., using the SF-36 questionnaire, reported remarkably good quality of life among older kidney transplant recipients [94]. In contrast, there were some who felt uncomfortable due to the prolonged recovery or medication-related side effects [95].

Jankowska et al. showed that even though the long-term patient and graft survivals were poorer among older subjects (aged 65 standard deviation years) versus younger subjects (aged 45 years), death-censored graft survivals were on the same level [96]. The main reason for graft loss in the elderly is the comorbidity-related demise of the patient [25]. This is why reliable research for comorbidities should be conducted. Such management can significantly minimize early post-transplant morbidity and mortality.

### 5.2. Prevention of Kidney Aging

Kooman et al. [44] have pointed out that an emerging factor affecting kidney aging is inflammation. An important aspect that affects inflammation is gut dysbiosis. It is not only associated with kidney dysfunction, but also with a number of disorders such as diabetes mellitus, colorectal cancer, inflammatory bowel disease, and even autism or Parkinson’s disease [97,98]. 

Diet has a great impact on the gut microbiome composition. For example, the Mediterranean diet (MD) is considered to have anti-inflammatory effects. These properties are attributed to an increase in Bacteroides and Clostridium phyla, and a decrease in Proteobacteria and Bacillaceae phyla [99]. The high-level consumption of plant-based food consistent with an MD may regulate the composition of bacteria in the gut and lead to beneficial changes in metabolomic profiles [100]. Moreover, microbiota-accessible carbohydrate supplementation can mitigate systemic inflammation in mice [101]. Borges et al. showed that probiotics can inhibit pathogenic bacteria proliferation, reduce the infammatory response and strengthen the permeability of gut mucosa [102]. On the other hand, it was reported that increased physical activity influenced bacterial diversity and the count for Bifidobacterium and Prevotella [103]. 

The MD has another beneficial effect. It belongs to diets with low acid production (like phosphates or sulfates). Products that have a high phosphate load are meat, dairy, or processed food. Sanchez-Roman et al. showed that eating meals rich in vegetables and fruits is associated with longer telomere length [104]. The Ornish diet (low-fat, plant-based) increased telomerase activity in men suffering from prostate cancer [105]. A low-phosphate diet is also considered to be beneficial for klotho deficiency. Stubbs et al. reported that it alleviated renal dysfunction and aging in mice [106]. In addition, it was found that the ketoacid analog supplementation of the low protein diet was effective in maintaining FGF23 and serum Klotho levels [107]. However, lack of this supplementation in this particular type of diet has led to increased levels of phosphate and decreased serum Klotho levels.

Another simple method for prolonging kidney conditions seems to be calorie restriction. McCay et al. demonstrated in their study that in rats subjected to life-long calorie restriction, median and maximal lifespan were noticeably prolonged [108]. Calorie restriction also has a beneficial effect on the age-associated FAO pathway impairment, as well as the kidney fibrosis process [59]. However, in humans, life-long calorie restriction is not achievable and may limit an active lifestyle. Nevertheless, even short-term calorie restriction can lead to a decrease in blood urea nitrogen, creatinine, and uric acid, as well as reduced blood pressure [109].

The findings of Sen et al. [110] suggest that SerpinB2, a serine protease inhibitor of the serpin superfamily, besides expressing in activated macrophages, is also upregulated in stressed tubular cells. The authors have shown that SerpinB2 promotes the proreparative adaptation of the kidney. As adaptive processes are critical in successful repair process and preserve kidney homeostasis during aging, SerpinB2 may be valuable for the research towards its role in kidney disease and human aging.

In addition, studies conducted on rats are promising. Benigni et al. showed that the blockade of the renin-angiotensin-aldosterone system may mitigate renal aging in those who suffered from HT [111]. Nonetheless, there is still a lack of studies showing the same correlation in humans.

Zhu et al. [112] demonstrated that senescent cells are susceptible to selective clearance by targeting pro-survival mechanisms using siRNAs and drugs. Furthermore, dasatinib and quercetin, prototype senolytic agents, have the ability to alleviate multiple aging phenotypes. Dasatinib is a short-acting inhibitor of multiple tyrosine kinases. It inhibits ABL, SRC, c-KIT, PDGFR-α, PDGFR-β, and ephrin receptor kinases [113]. Quercetin is responsible for the promotion of the loss of cell viability, apoptosis and autophagy through the modulation of PI3K/Akt/mTOR, Wnt/-catenin, and MAPK/ERK1/2 pathways [114].

Autophagy is an intracellular degradation system that has been proven to protect kidneys from inflammatory insults which are strictly related to aging [115]. Its major function is to remove excess long-lived proteins and damaged organelles [116]. Nevertheless, it has been demonstrated that autophagy decreases with age [117]; hence, the accumulation of cellular damage leads to the aging process. Cui et al. reported the remarkably decreased expression of autophagy markers—autophagy-related gene (Atg)7 and LC3/Atg8— in aged rat kidneys [118]. Dysfunction in autophagy is also associated with the pathomechanism of many diseases, e.g., glomerulosclerosis or renal ischemia/reperfusion injury [119,120]. Due to this fact, therapeutic options targeting autophagy appear to be a key element in the management of renal aging. However, it should be remembered that this type of treatment also has side effects. Too massive and rough modulation of autophagy can contribute to these side effects. For example, chloroquine, an autophagy inhibitor, has been demonstrated to promote chemotherapy-induced kidney injury [121].

### 5.3. The Effects of Resveratrol on Kidney Aging

Recently, resveratrol (3,4′,5-trihydroxy-trans-stilbene) has emerged as a potential anti-aging agent. This phenolic compound is commonly found in plants, especially in red grapes. It owes its antioxidant effect to the redox properties of its phenolic hydroxyl group and the potential for electron delocalization across the conjugated structure [122]. Tung et al. have shown that resveratrol, combined with exercise, reversed the effect of aging in old mice [123]. 

Uddin, in his literature search, has shown that resveratrol is a factor that directs aging biomarkers, such as SIRT1, 5’ AMP-activated protein kinase (AMPK), and nuclear factor-κB (NF-κB), to the protection of the kidneys [124]. Moreover, due to the inhibition of the formation of reactive oxygen species, resveratrol has proven its potency against AKI in humans [125]. It was also shown that resveratrol can increase cell viability by reducing the phosphorylation of NF-κB, as well as the accumulation of inflammatory factors in response to lipopolysaccharide [126].

However, the dose is a very crucial issue. While resveratrol at a lower dose can be very beneficial in maintaining human health, higher doses have a pro-apoptotic effect—thus, it is dangerous for healthy cells, but it can be used in anti-cancer treatment [127].

It is worth noting that most findings were mostly based on preclinical studies. The clinical applications of resveratrol are limited due to its low bioavailability and limited durability.

## 6. Conclusions and Perspectives

Renal aging is a complex process having an impact on all components of the kidney and leading to a loss of kidney function. The main macroscopic changes in aging kidneys are a reduction of a kidney mass, a decrease in the volume of renal parenchyma with its further calcification, renal artery fibromuscular dysplasia, atherosclerotic narrowing, focal cortical thinning, and more. Among histological changes, it is possible to distinguish the thickening of the glomerular basement membrane, nephrosclerosis, the accumulation of extracellular matrix, and mesangial widening. GFR also declines with normal aging. Several microscopic and macroscopic changes in aging kidneys are necessary to assess the progression of the process, but overlapping comorbidities affecting the elderly could make this challenging. There are several non-modifiable and modifiable risk factors such as gender, race, CVD, HT, DM, smoking, obesity, and high protein intake that all contribute to aging by accelerating it, and, in some cases, even leading to premature death. A vital factor that also should be taken into consideration in renal aging is oxidative stress mediated by SIRT1, PGC-1α, PPARα, and Klotho. As renal senescence is associated with the risk of AKI and CKD, special care should be taken for the elderly who are most at risk of these diseases. In some cases, dialysis or even kidney transplantation is necessary.

Some studies highlight the beneficial effects of the Mediterranean diet, restriction of meat consumption, and calorie restriction in general on the functioning of the kidneys and the whole organism.

Despite the characteristic changes in the functioning and appearance of the kidneys being wholly assumed, the exact mechanisms of kidney senescence are still uncertain. It is challenging to distinguish between changes in the physiological aging of the kidneys and those in age-related kidney diseases and comorbidities. Because the kidneys are very susceptible to the aging process, and the condition of the kidneys is one of the predictors of longevity, it is crucial to recognize any dysfunction early and implement appropriate treatment. Understanding the course of these processes and developing ways to slow down renal aging may extend health span and life. Research on the nephroprotective role of PPARγ agonists and SerpinB2 activity is critical. Since there are reports that resveratrol has emerged as a potential anti-aging agent, further research on this compound is necessary.

## Figures and Tables

**Table 1:** Types of cell senescence in kidney [31].

Cells	Markers of Cellular Senescence	Models	References
proximal tubules	SA-β-Gal (EM)/p16Ink4a, p19Arf, p21Cip1 (qPCR)	mouse	[36]
cortical tubules	SA-β-Gal, p16INK4a (IHC/qPCR), p19ARF (qPCR)	mouse, rat	[37]
	p16INK4A, p27KIP1 (IHC)	human	[38]
cortex, glomeruli, intersitium, arteries	p16INK4A, TP53 (IHC)	human	[39]
	p16INK4A, TP53, TGFβ1 (IHC)		
	p16INK4A, TP53, p14ARF, TGFβ1 (IHC)		

**Table 2:** Pharmacotherapeutic targets in DKD and the aging kidney [60].

Compounds	Intervention Strategy	Models	References
SIRT1	activating Nrf2/ARE pathway	GMCs	[61]
osthole	inducing klotho expression	human renal proximal tubular cells	[62]
irbesartan	suppressing RAGE expression	proximal tubular cells	[63]
gliclazide	inhibiting RAGE-p22phox-NFκB pathway	GMCs and renal tubular epithelial cells (HK-2)	[64]
fluorofenidone	blocking RAGE/AGEs/NOX and PKC/NOX pathways	db/db DN mice	[65]
loganin and catalpol	blocking AGEs–RAGE signaling	DN rats	[66]

## Data Availability

The data used in this article are sourced from materials mentioned in the References section.
